# Intraoperative changes of surgical approach and a second surgery after percutaneous endoscopic surgery for lumbar spinal stenosis

**DOI:** 10.1016/j.clinsp.2024.100498

**Published:** 2024-09-28

**Authors:** LianSong Lu, ZhenShan Yuan, HaoJie Li, ShaoHua Sun

**Affiliations:** Department of Spinal Surgery, Ningbo No.6 Hospital, Ningbo City, Zhejiang Province, China

**Keywords:** Lumbar spine, Stenosis, Endoscopy, Surgery, Outcomes, Lumbar Intervertebral Disc Herniation

## Abstract

**Objective:**

To investigate the reasons for a second surgery after Percutaneous Endoscopic Surgery (PES) for lumbar spinal stenosis and to provide references for the choice of indications and appropriate surgical approach.

**Method:**

A total of 426 patients received PES for lumbar spinal stenosis. The postoperative, intraoperative, and postoperative data of the subjects were analyzed. The reasons for intraoperative difficulties, poor outcomes after surgery, and a second surgery were analyzed.

**Result:**

The surgical approach was changed in four out of 426 patients (0.94 %) during surgery, and 6 patients (1.4 %) received a second surgery; 3 out of 4 patients were intraoperatively shifted to PIED using the Delta endoscope, and 1 shifted to ordinary PIED. The reasons for the intraoperative change of surgical approach included severe hyperplasia and obscure anatomic structure in 3 patients and a dural sac tear with neural outflow in 1 patient. The reasons for a second surgery in 19 patients were as follows: nerve entrapment by bone fragments in 1 patient; nerve injury in 3 patients; lumbar instability in 3 patients; untreated or residual Lumbar Intervertebral Disc Herniation (LIDH) in 4 patients; recurrent LIDH in 1 patient; and inadequate decompression in 7 patients.

**Conclusion:**

Severe hyperplasia, obscure anatomic structure, lumbar instability, and nerve injury are the common reasons for a second surgery after PES for lumbar spinal stenosis. Appropriate indications and surgical approaches can be chosen based on the patient's situations and technical conditions.

## Highlights

• Surgical approach changes due to severe hyperplasia, obscure anatomic structure, etc.
• A second surgery is due to nerve entrapment, nerve injury, lumbar instability, etc.
• Appropriate indications and surgical approaches are chosen accordingly.

## Introduction

In recent years, the authors have witnessed population aging and increasing prevalence of lumbar spinal stenosis, and surgical treatment may be indicated for some severe patients. Microendoscopic Discectomy (MED) can remove the hyperplastic compression-inducing lesions in the posterior areas and achieve adequate decompression. With Mobile Microendoscopic Discectomy (MMED), the working channel can be tilted at any angle, thereby making decompression more convenient.^1^ Because of this benefit, MMED has become a common technique for treating lumbar spinal stenosis. Rapid progress has been made in Percutaneous Endoscopic Surgery (PES) and Full Endoscopic Surgery (FES), including the techniques of Percutaneous Transforaminal Endoscopic Discectomy (PTED) and Percutaneous Interlaminar Endoscopic Discectomy (PIED). The indications have been expanded from simple Lumbar Intervertebral Disc Herniation (LIDH) to lumbar spinal stenosis, with good outcomes achieved. Many scholars believe that PES has smaller incisions and less invasiveness with a more thorough exposure to the surgical field in a water medium. PES has the potential to replace MED and MMED.^2^, ^3^, ^4^, ^5^ The authors have also encountered some cases where intraoperatively shifted to MMED from EPS for lumbar spinal stenosis but later required a second surgery due to poor outcomes. This points to the limitations of PES. The present study was concerned with the reasons and preventive measures for intraoperative changes of surgical approach from PES or a second surgery after PES. The purpose was to provide references for the choice of indications and surgical approach.

## Materials and methods

### Indications and contraindications for primary PES for lumbar spinal stenosis

Indications: (1) Lower back pain with radiating pain, numbness or intermittent claudication in the lower extremities, especially on one side; (2) Oswestry Disability Index (ODI) > 30 %, and Visual Analogue Scale (VAS) > 5; (3) Poor response or relapse after over 3 months of conservative treatment, which affected the patients’ work and daily life; (4) Apparent lumbar spinal stenosis upon Computed Tomography (CT) and Magnetic Resonance Imaging (MRI), including stenosis of lateral recess and intervertebral foramen on one side, with or without LIDH, which were consistent with clinical manifestations and signs.

Contraindications: (1) Severe lumbar spinal stenosis or obstruction, severe symptoms, and cauda equina syndrome in bilateral lower extremities; (2) Severe bony lumbar spinal stenosis, with anticipated difficulties of decompression by PTED; (3) Active lumbar spondylolisthesis or lumbar instability upon the extension and flexion views (angulation > 15°or displacement > 3.5 mm); (4) Lumbar spine fracture, infection and tumors; (5) Multi-segment LIDH with lumbar spinal stenosis, presenting with symptoms inconsistent with the radiologic examinations; (6) Mental abnormalities, drug abuse and communication difficulties.

### Basic information

From January 2015 to January 2019, 426 patients received PES for lumbar spinal stenosis at the department, including 305 patients receiving the ordinary PTED and 100 patients receiving PIED using the Delta endoscope. Among them, 4 patients (0.94 %) were intraoperatively shifted to MMED, and 6 patients (1.4 %) underwent a second surgery due to poor outcomes or relapse following the primary surgery. There were 13 patients with lumbar spinal stenosis who received secondary surgery at the department due to poor outcomes following PES at other hospitals. Therefore, there were 23 patients included in the present study. Among them, there were 12 males and 11 females, who were aged 51 to 82 years with an average of 67.2 years. As to the segments treated by primary PES, 1 patient was operated in L_3–4_ space, 14 patients operated in L_4–5_ space, 3 patients operated in L_5_-S_1_ space, 2 patients operated in L_3–5_ space, and 3 patients operated in L_4_-S_1_ space; 14 patients were treated by primary PTED, 5 patients by PIED using Delta endoscope, and 4 patients by ordinary PIED.

## Methods

### Analysis

Preoperative clinical manifestations and radiological features of the patients who were intraoperatively shifted from PES were analyzed, and outcomes after surgery and at the follow-up were recorded. Surgical approaches before and after intraoperative shifts were also recorded. The reasons for intraoperative shift were analyzed.

Preoperative and postoperative data for the primary PES and second surgery were analyzed, including clinical manifestations and radiological changes. The segments operated by the primary surgery, approach of the primary surgery, approach of the second surgery, intraoperative findings during the second surgery, and postoperative follow-up were statistically analyzed. The reasons for the second surgery were analyzed.

### Evaluation indicators and criteria

The patients were followed up for 6‒36 months after the intraoperative shift or the second surgery, with an average of 18.2 months. VAS scores and ODI before the second surgery and during the follow-up were evaluated, and the outcomes were analyzed using the Macnab criteria: excellent, the symptoms completely disappeared, and the patients could not restore normal life and work activities; good, the patients had occasional pain and could do less laborious work; fairly good, the symptoms were alleviated despite persisting pain, and the patients could not work; poor, the patients had nerve root compression and required further surgical treatment.^6^ This study follows the STROBE statement.

## Results

### Intraoperative shift

Among 4 patients undergoing an intraoperative shift, 3 subjects were shifted to bilateral decompression via unilateral fenestration by PIED using Delta endoscope, and 1 shifted to unilateral decompression with discectomy by PIED. The primary reasons for the intraoperative shift below were analyzed:

1) Obscure anatomic structure and decompression difficulties:

Case 1 had lumbar spinal stenosis in L_4,5_ space, with severe hyperplasia, vertebral plate thickening, facet joint hypertrophy, and deformation. In the primary PIED using the Delta endoscope, the anatomic structure and boundary were not clearly shown, and it was difficult to locate the interlaminar space and medial border of the articular process. This patient was intraoperatively shifted to MMED to enlarge the exposure and to differentiate the anatomic structure. After grinding the hyperplastic osteophyte, the interlaminar space was fully exposed and the surgery was successfully performed.

**Fig. 1:**
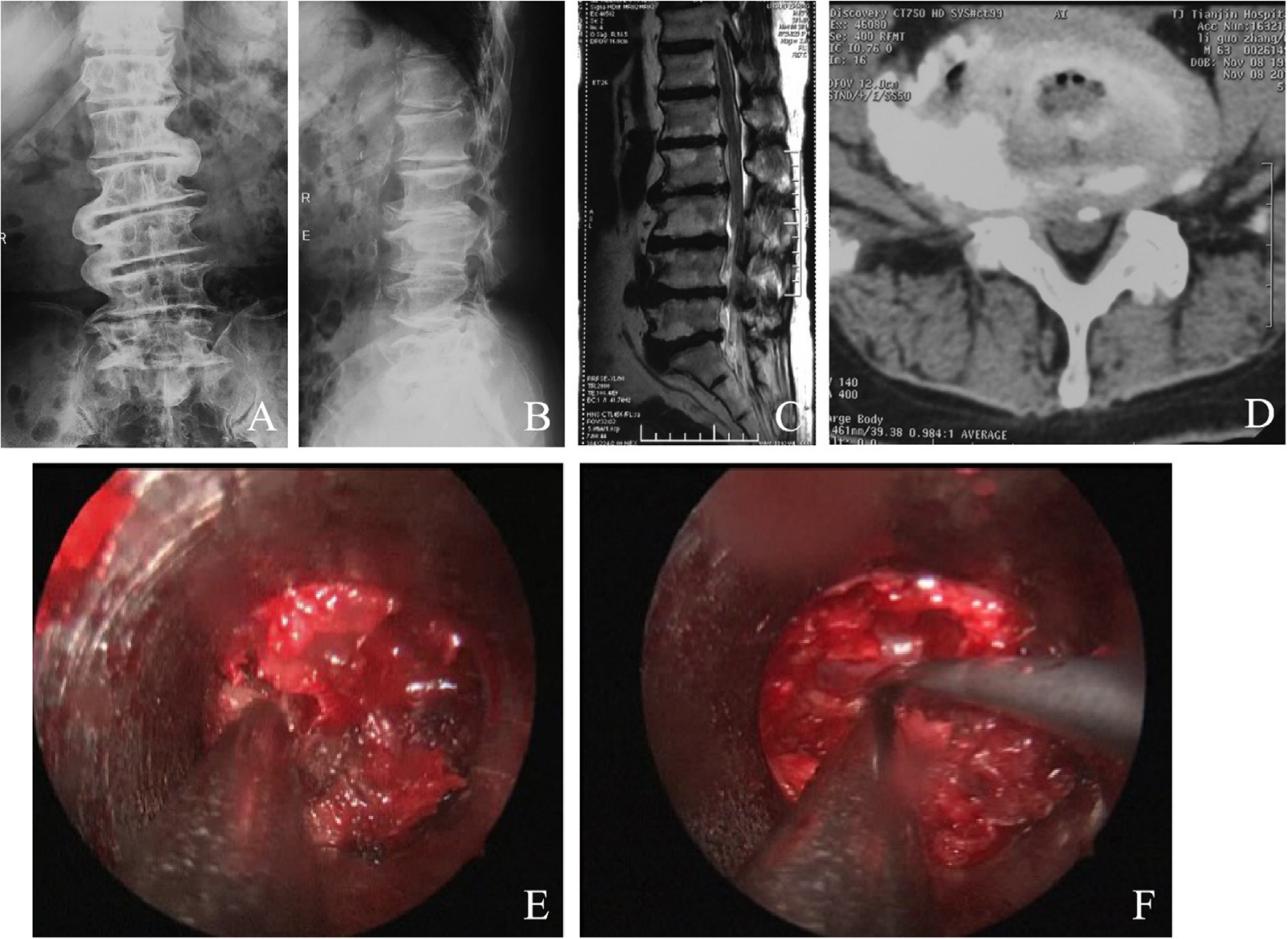
Case 2, a male patient aged 63 years, with lumbar spinal stenosis in the L_3–5_ space. (A‒B) Preoperative anteroposterior and lateral X-Ray revealed severe lumbar vertebral osteoporosis. (C‒D) Preoperative MRI revealed LIDH and severe lumbar spinal stenosis in the L_3–5_ space. PIED was performed first. The anatomic structure was not clearly visualized, with loss of orientation. This patient was intraoperatively shifted to MMED with adequate decompression, loosening of the dural sac and nerve root.

Case 2 had lumbar spinal stenosis in L_3–5_ space, with severe hyperplasia. In the primary PIED, the hyperplastic osteophyte, thickened vertebral plate and inferior articular process were drilled away. However, due to severe hyperplasia, the anatomic structure was not clearly shown, resulting in the loss of orientation and difficulty in determining the location and scope of decompression. This patient was intraoperatively shifted to MMED. It was later revealed that the decompression was inadequate in the previous PIED. After expanding the decompression zone, the dural sac and nerve root were exposed. Thus, adequate decompression was achieved, and the surgery was successfully performed (Fig. 1).

Case 3 had lumbar spinal stenosis in L_5_S_1_ space with LIDH, ossification, and herniation of nucleus pulposus tissues. The free nucleus pulposus tissues were found in the subaxillary region between the dural sac and nerve root. PIED failed to fully expose this region, and resection of nucleus pulposus tissues may cause a rupture of the medial nerve root. This patient was intraoperatively shifted to MMED to remove the distal free nucleus pulposus tissues. After extended decompression, the partial defect was observed in the subaxillary region of the nerve root sleeve. This patient had numbness in the affected limb after surgery, which was improved within 1 week.

2) Dural sac tear with neural outflow:

Case 4 had lumbar spinal stenosis in L_4,5_ space, and the stenosis was most severe in this case. After sclerotin grinding, the dural sac was torn during the resection of the ligamentum flavum under the endoscope. As a result, the cauda equine came out. After lowering the water pressure, it was difficult to perform a reduction of the nerve, which further influenced the subsequent operation. This patient was intraoperatively shifted to MMED. Following the reduction of the cauda equine, the nerve was protected with a cotton pad, and bilateral decompression was performed. The symptoms were improved after surgery, without injury of the cauda equine. The outcomes were good according to the Macnab criteria.

### A second surgery

Among 6 patients receiving a second surgery at the studied department, 4 patients were treated by PTED, and 2 patients by PIED using the Delta endoscope. Among 13 patients receiving a second surgery following the primary surgery at other hospitals, 10 patients were treated by PTED and 3 patients by PIED. The major reasons for a second surgery were as follows:

1) Nerve entrapment by bone fragments:

Case 5 had lumbar spinal stenosis in L_4,5_ space with LIDH. The radiating pain in the affected lower limb was aggravated soon after receiving PTED at the studied department. The symptoms were not relieved by conservative treatment, including bed rest, intravenous use of hormones, and dehydration drugs. MIR revealed mixed signals in the L_4,5_ space, which were suspected of nerve root compression. MMED exploration was performed on the next day after the primary surgery. It was found that the dural sac was entrapped by a small irregular piece of bone fragment in the lateral recess. After removing the bone fragment, there was a tear in the lateral and dorsal sides of the dural sac, with Cerebrospinal Fluid (CSF) leak and outflow of the nerve fibers. The small bone fragment might be produced by resection of the articular process using the trephine during PTED. The small bone fragment was displaced towards the dorsal side and penetrated into the dural sac. Since the working channel in PTED was located on the ventral side of the dural sac, the small bone fragment was beyond the working channel and visual field. Moreover, the CSF leak was masked by the high pressure of the water medium, resulting in the failure to discover such a condition in PTED. Compression by the bone fragment was relieved under MMED. The dural sac tear was small and was not sutured. Decompression was adequately performed along the nerve root, and the symptoms were relieved afterward. The outcomes were good according to the Macnab criteria during the last follow-up.

2) Nerve injury:

Cases 6 and 7 were patients who had received PIED at other hospitals. Case 6 reported alleviation of pain in the buttock after surgery, although there was still skin numbness in the dorsum of the foot and weakness in the dorsal extension of the toes on the affected side. This patient suffered from numbness and aggravated pain in the lower limbs at 1 month after surgery. Case 7 reported weakness in the dorsal extension of the toes, skin numbness, and pain in the dorsum of the foot, with intermittent claudication. These two patients were reexamined by CT and MR after surgery, both of which revealed inadequate decompression and apparent lumbar spinal stenosis. In order to better restore the neural functions, open surgery was performed for total vertebral plate resection and decompression, intervertebral fusion, and internal fixation. Intraspinal stenosis and severe adhesion were observed. Adequate loosening of the nerve was administered along the nerve root canal. Both two cases reported alleviation of the symptoms after surgery, with partial improvement of the tone of the toe extensor. The outcomes were fairly good according to the Macnab criteria during the last follow-up.

Case 8 had lumbar spinal stenosis in L_4_-S_1_ space. After receiving PTED at other hospitals, this patient-reported symptoms of incomplete injury of the L5 nerve root. This patient received open revision surgery at the department 2 weeks after the primary surgery, which revealed a contusion of the nerve root in double segments with rupture of part of the nerve fibers. The symptoms were partially relieved after surgery, and the outcomes were fairly good according to the Macnab criteria in the last follow-up.

**Fig. 2:**
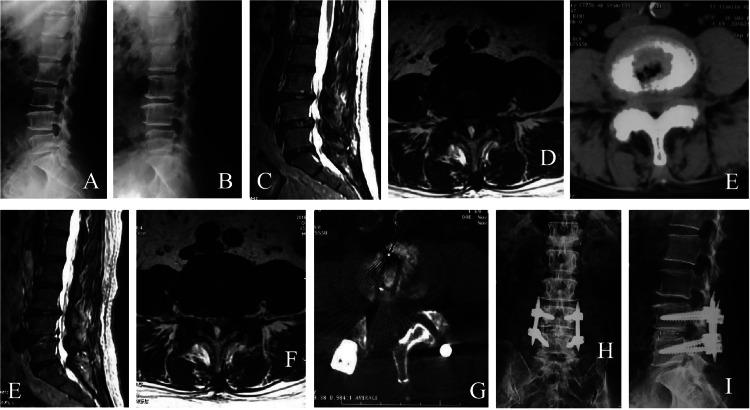
Case 9, a female patient aged 67 years, with lower back pain, right lower extremity pain, and intermittent claudication for 4 years. (A‒B) Lumbar extension-flexion X-Ray showed grade I unstable spondylolisthesis in L_4,5_ space. (C‒D) Sagittal MRI showed lumbar spinal stenosis and LIDH; cross-sectional MRI showed spinal stenosis in L_4,5_ space. (E) CT showed that the spinal stenosis was most severe in L_4,5_ space; 270°PTED was performed to the ventral, lateral and dorsal sides of the nerve, and the nerve was obviously relaxed under the endoscope. The symptoms were temporarily relieved after surgery, but the symptoms were aggravated again after getting out of bed. (F‒G) MRI sagittal T2-weighted images showed hyperintensities in L_4,5_ space, and cross-sectional images still indicated spinal stenosis. Decompression and interbody fusion+ percutaneous fixation were performed with MMED. Symptoms were significantly relieved after surgery. (H) The postoperative CT scan showed adequate decompression; anteroposterior and lateral views of the lumbar spine at 1-year after surgery. (I) Showed good fusion and fixation, with excellent outcomes.

3) Lumbar instability:

Cases 9, 10 and 11 reported alleviations of the symptoms after PTED in L_4,5_ space at the studied department. The symptoms were aggravated at 2, 5 and 14 days after surgery, respectively, especially in standing position and during movement. They responded poorly to the conservative treatment. Their radiologic data were reviewed. Lumbar instability and degeneration and unstable spondylolisthesis were found on the extension and flexion X-Ray (active spondylolisthesis on dynamic radiographs). In PTED, decompression was performed for the dural sac and ventral, lateral, and dorsal sides of the nerve root (270° decompression). Adequate decompression was provided, and the nerve root became relaxed without tension. However, postoperative radiographs still indicated lumbar spinal stenosis and lumbar instability (Fig. 2). These 3 patients received another surgery at 2 to 4 weeks after surgery. Case 9 received open decompression, interbody fusion and fixation; Case 10 and 11 received decompression, interbody fusion + percutaneous pedicle screw fixation under MMED. All of them achieved significant symptom improvement after surgery. The outcomes were excellent in 1 case and good in 2 cases according to Macnab criteria during the last follow-up.

4) Untreated or residual LIDH:

Cases 12, 13, 14 and 15 had already received PTED at other hospitals due to lumbar spinal stenosis with LIDH. The symptoms were alleviated significantly at the beginning. However, lower back and leg pain recurred when getting out of bed or during gentle waist twisting at 1‒3 weeks after surgery. Reexamination by MRI revealed LIDH, nerve compression and edema of the surrounding tissues. The patients poorly responded to the conservative treatment, and all of them received a second surgery at the studied department. Two of them received PTED and the other two were MMED. Massive, degenerated nucleus pulposus was found in the dural sac and ventral side of the nerve root, compressing the nerve. The loose intervertebral disc tissue mass was removed with adequate decompression, and the symptoms were significantly alleviated after surgery. The outcomes were excellent in 1 patient, good in 2 patients, and fairly good in 1 patient at the last follow-up according to the Macnab criteria.

**Fig. 3:**
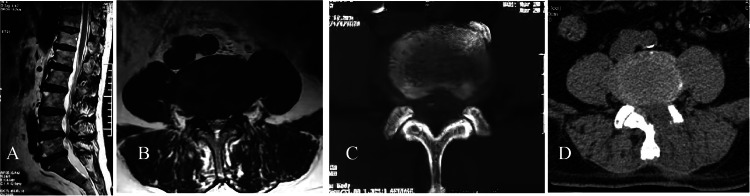
Case 16, a male patient aged 73 years, with lower back pain, left lower extremity pain and intermittent claudication for 5 years. (A‒B) Preoperative MRI showed lumbar spinal stenosis in L_4,5_ space and LIDH. (C) After the first surgery, CT showed that the herniated intervertebral disc tissue compressed the nerve. (D) After MMED, CT showed adequate decompression.

Case 16 had lumbar spinal stenosis in L_4,5_ space with LIDH. The symptoms were partially alleviated after PIED using a Delta endoscope. However, the symptoms were aggravated on the next day after surgery to the preoperative level. The patient poorly responded to the conservative treatment using hormones and dehydration, and the nerve root symptom was conspicuous. Reexamination by CT revealed nerve compression by the intervertebral disc tissues in the lateral recess. MMED exploration was performed via the original incision 5 days later, which revealed nerve root compression with high tension in the lateral recess. The superior margin of the L_5_ vertebral plate was resected for extended decompression, and the nerve root was pushed medially. The herniated and free intervertebral disc tissues on the ventral side were removed, thereby achieving adequate decompression of the nerve root. The patient's symptoms were alleviated after surgery (Fig. 3), and the outcomes were good at the last follow-up according to the Macnab criteria.

Case 17‒21 had previously received PTED at other hospitals due to lumbar spinal stenosis, but achieved poor outcomes. The symptoms were not significantly improved in Case 17, and were further aggravated after partial remission in Case 18, 19 and 20; Case 21 suffered from more severe symptoms after surgery than before. Postoperative CT and MRI indicated significant lumbar spinal stenosis and nerve compression in the previously operated segments, and signs of partial decompression were only shown around the catheterization channel. This was considered relevant to poor surgical outcomes and small decompression scope. In particular, the working channel of PTED was unparallel with the intervertebral space on the L_5_S_1_ level, where the operation space was limited, thus further reducing the decompression. The outcomes of postoperative conservative treatment were still unsatisfactory in these cases, who later received revision surgery with MMED at the hospital 1‒5 months after the primary surgery, respectively. The medial aspect of the articular process and the hyperplasic and thickened ligamentum flavum lateral to the spinal canal were removed during the revision surgery. Adequate decompression was performed along the working channel of the nerve root, removing the herniated and fragmented intervertebral disc tissues. All of the five cases achieved significant symptom improvement after the revision surgery. At the last follow-up, the outcomes were excellent in 2 cases and good in 3 cases according to the Macnab criteria.

Case 22 had received PIED at other hospitals due to lumbar spinal stenosis in L_4,5_ space, but the lower back and leg pain was aggravated after surgery. Reexamination by MRI indicated hyperintensities in the decompression region on the MRI T2-weighted images. This was attributed to apparent edema and inadequate decompression. Since this patient was combined with mild lumbar instability in L_4,5_ space, a secondary open surgery was performed. Intraoperative findings included apparent adhesion around the nerves, which was treated by fusion and fixation following adequate decompression. The symptoms were significantly improved after the revision surgery. The outcomes were excellent at the last follow-up according to the Macnab criteria.

Case 23 previously received decompression in L_4,5_ space by PIED using a Delta endoscope at the department. The symptoms were improved at 3 days after surgery. However, lower back and leg pain was aggravated afterward, and the patient did not respond to the conservative treatment. Reexamination by MRI indicated inadequate decompression and residual lumbar spinal stenosis. A second MMED was performed at 2 months after surgery for adequate nerve decompression. The symptoms were significantly improved after the second surgery. The outcomes were excellent at the last follow-up according to the Macnab criteria.

The main reasons for a second surgery following PES in 19 cases were summarized as follows: nerve entrapment by free bone fragments with dural sac tear in 1 case; nerve injury in 3 cases; lumbar instability in 3 cases, LIDH in 5 cases, including residual or recurrent herniation; and inadequate decompression in 7 cases. Approaches for the second surgery included PTED in 2 cases, MMED in 10 cases, MMED with microinvasive fusion and fixation in 2 cases, and open decompression with fusion and fixation in 5 cases. All the patients achieved significant improvement after the second surgery.

The VAS score for leg pain among 23 patients who were intraoperatively shifted or received a second surgery decreased from 7.2 ± 3.8 before the second surgery to 1.9 ± 2.1 at the last follow-up; the VAS score for the lower back pain decreased from 6.9 ± 3.4 before the second surgery to 2.1 ± 1.9; ODI decreased from 36±15 before the second surgery to 7.9 ± 6. According to the Macnab criteria, the outcomes were excellent in 6 cases, good in 11 cases, and fairly good in 6 cases.

## Discussion

### Advantages and limitations of PES for treating lumbar spinal stenosis

Lumbar spinal stenosis is generally caused by degenerative factors, including intervertebral disc degeneration, bulge or herniation, ligamentum flavum hypertrophy, vertebral plate thickening, articular process hypertrophy and deformation. These conditions are more likely to happen on the basis of developmental lumbar spinal stenosis. Therefore, hyperplasia and degeneration of the posterior structures are important pathogenic factors. Depending on the site of stenosis, lumbar spinal stenosis is divided into central and lateral types. Compared with MED or MMED, PES was less invasive for the treatment of lumbar spinal stenosis. It is usually performed via three approaches, namely, the transforaminal approach in PTED, the interlaminar approach in PIED, and the posterior approach in PIED using a Delta endoscope.

PTED has been applied most extensively with the intraoperative use of local anesthetic and a skin incision of only 8 mm. After the puncture, the soft tissues are dilated stepwise using the sleeve. Following foraminotomy, the spinal canal is accessed via the intervertebral foramen. Continuous lavage is provided with a water medium during the operation to reduce bleeding, enable a clear surgical field, and cause less trauma. The intervertebral disc, and lateral and dorsal sides of the ligamentum flavum are resected under the endoscope. The 270° decompression is performed around the dural sac and nerve, and the desired outcomes can be achieved. This method is especially suitable for elderly patients who are poorly tolerant to general anesthesia or open surgery.^7^^,^^8^ However, PTED also has its limitations. The compression causing lumbar spinal stenosis usually comes from the posterior structure. With the working channel created via the transforaminal approach, the surgical field is closer to the intervertebral disc and it is difficult to fully expose the posterior spinal canal. In order to provide decompression to the posterior spinal canal, resection of the articular process and foraminotomy are needed to access the posterior spinal canal. However, this places a high requirement on the catheterization skills of the surgeons and the surgical procedures are complex. For mild to moderate lateral stenosis or stenosis featured by ligamentum flavum hypertrophy, PTED can provide decompression to the lateral and dorsal sides of the nerve root by stepwise resection of the hyperplastic ligament. However, this procedure can hardly deal with bony hyperplasia and usually requires grinding under the endoscope or higher skills in the cutting of sclerotin. It is even more difficult to perform decompression to the central and lateral stenosis using this method. Excessive resection of the articular process during decompression will impair lumbar stability, while excessive squeezing of the dural sac will cause nerve injury. Moreover, hyperplasia is usually extensive at the site of lumbar spinal stenosis, thereby blocking the working channel and making adjustment and exposure to the surgical field difficult. So precise intraoperative localization and stepwise dilation are needed to achieve adequate decompression.^9^

PIED uses the posterior interlaminar approach for decompression, which can directly remove the ligamentum flavum and medial border of the hyperplastic and hypertrophic articular process that causes nerve compression. This is conducive to fully exposing the nerve root and dural sac, thereby achieving sufficient decompression. Lumbar spinal stenosis is usually accompanied by severe hyperplasia. To increase the decompression efficiency, the use of a Delta endoscope is preferred along with efficient grinding of the hyperplastic and thickened inferior border of the vertebral plate and medial aspect of the facet joint. A large-bite Kerrison rongeur is used for the resection of the hypertrophic ligamentum flavum to achieve adequate decompression. A narrower working channel can be inserted via the larger working channel if necessary to ward off the nerve root and dural sac, so as to better expose the intervertebral sac and to resect the herniated intervertebral disc. The larger working channel currently in use for the Delta endoscope has a diameter of about 1 cm. Compared with MMED, the exposure is more limited. Articular process hypertrophy and deformation in lumbar spinal stenosis will cause narrowing of the interlaminar space. In some serious cases, obscure differentiation of anatomic structure as well as loss of position and orientation will occur. Besides, a larger working channel will also cause a reduction in water pressure. Given the large number of bleeding points in lumbar spinal stenosis, hemostasis must be performed frequently, leading to a greater production of debris, which further influences the surgical field. General anesthesia is also needed in most situations, which increases the anesthesia-related risks.

### Reasons and preventive measures for intraoperative shift and a second surgery following PES for lumbar spinal stenosis

In the present study, intraoperative difficulty and shift were encountered during PES for lumbar spinal stenosis in 4 cases. There was severe hyperplasia at the lesion site in 2 cases. After grinding away part of the sclerotin under PIED using the Delta endoscope, the local anatomic relationship became hard to differentiate, resulting in the loss of position and orientation. In one case, the difficulty of resection and decompression was encountered due to LIDH and free nucleus pulposus tissues in the subaxillary region; in another case, the surgical field was affected by dural sac tear and neural outflow, resulting in the intraoperative shift to MMED. The above facts indicate that severe hyperplasia and facet joint deformation will interfere with the creation of working channels and endoscopic manipulations in PIED. Timely localization or intraoperative shift is needed in case of obscure anatomic relationships. After accessing the spinal canal, LIDH with large ossification will also influence manipulations and exposure, resulting in the difficulty of performing resection of the nucleus pulposus tissues and decompression. Dural sac tear during the resection of the hypertrophic ligamentum flavum or hyperplastic sclerotin may lead to neural outflow, thereby influencing the surgical field. Moreover, a higher water pressure can also increase the intracranial pressure. Therefore, the surgeons need to keep away from the nerves and finish the decompression as quickly as possible, or choose an intraoperative shift if indicated.

There were 19 patients undergoing a second surgery after PES for lumbar spinal stenosis, for the following reasons: displacement of free bone fragments, nerve injury, lumbar instability, LIDH and inadequate decompression.

1) Nerve entrapment by free bone fragments: Case 5 was not found with dural sac tear and bone fragment displacement during the primary PTED until the second surgery was performed with MMED. The origin of the bone fragments and the reasons for the initial failure to discover the bone fragments were first analyzed, and preventive measures were developed accordingly. Before creating the working channel in PTED, foraminoplasty needs to be performed first, by using the trephine or grinding away part of the articular process. This process will generate debris or small free bone fragments. If the bone fragments enter the spinal canal, they may be pushed towards the dorsal side of the dural sac during the creation of the working channel. As a result, the bone fragments are beyond the working channel and surgical field, which makes the discovery of bone fragments difficult during PTED. This patient was not found with a dural sac tear during PTED, which was probably due to local packing and compression by the bone fragments. The water medium continuously perfused also exerted a certain pressure, which helped conceal the CSF leak or neural outflow at the site of the dural sac tear. All these factors leaded to the failed discovery of the free bone fragments. Therefore, during PTED, especially in those with severe hyperplasia, the resected tissues during foraminoplasty should be timely taken out. The patients’ response should be clearly observed during surgery. X-Ray scans can be performed to adjust the position and orientation of the trephine or working channel if there are signs of neural stimuli. Before ending the surgery, it should be carefully observed at all angles to avoid residual compression.
2) Nerve injury: Two patients who had previously received PIED at other hospitals suffered from traversing nerve root injury, and another patient who had received PTED at other hospitals presented with exiting nerve root injury. The reasons may be direct or indirect injury caused by surgical equipment or manipulations to the nerve. Lumbar spinal necrosis can narrow the space of the nerve and dural sac, reducing their mobility. In PTED or PIED, the use of trephine, dilation tube and working channel may exert further pressure on the nerve that is already compressed, thus aggravating nerve injury. In addition, lumbar spinal stenosis is usually accompanied by anatomic changes in joints or degenerative lumbar scoliosis due to hyperplasia. This will reduce the room available for equipment manipulation. Forceful insertion of the devices may cause nerve injury. Blind manipulation under general anesthesia without neurological monitoring may also cause damage to the exiting nerve root. To overcome these defects, PTED is performed under local anesthesia with close monitoring of the patients’ response or neurological monitoring. Before foraminoplasty using the trephine and bone drill, the position and direction of the devices should be confirmed by anteroposterior and lateral X-Ray to avoid mistaken insertion of the device into the spinal canal, causing nerve injury. If necessary, a drill can be used for foraminoplasty with careful discrimination to avoid damage to the compressed nerve.^10^^,^^11^
3) Lumbar instability: All the 3 patients undergoing a second surgery had previously received PTED. Although PTED can access the ventral, lateral and dorsal sides of the nerve for 270° decompression, the lower back and leg pain was still aggravated after the second surgery in 3 patients. The radiographs were reviewed, and it was found that all of them were already combined with lumbar instability before surgery. Intraoperative articular process arthroplasty and resection of the annulus fibrosus of the posterior intervertebral disc can further influence the stability of the involved segments. Although adequate decompression of the nerve channel can be achieved by PTED, the exposure of this approach is still limited. The pressure created by continuous perfusion of the water medium may conceal nerve compression, resulting in inadequate decompression and residual nerve compression. The symptoms will be further aggravated under the dual actions of deteriorating lumbar instability and inadequate decompression. In the present study, the symptoms were relieved in all of them after the second surgery for complete decompression + interbody fusion. This also proves from another perspective that lumbar instability is an important factor inducing the relapse. Since PES cannot provide appropriate treatment for lumbar instability, this procedure should be chosen with caution for lumbar spinal stenosis with lumbar instability.
4) Untreated or recurrent LIDH: The symptoms were relieved or partially relieved after PES in 5 patients, but relapse occurred after waist movement. MRI indicated apparent LIDH. The second surgery confirmed the residual herniated intervertebral disc tissues in the previously operated segments or reemergence of massive, herniated nucleus pulposus tissues and loose intervertebral disc tissues at the original site of operation. Reasons for the second surgery included incomplete resection of the herniated intervertebral disc, resulting in residual or recurrent herniation. The above facts suggest that when treating lumbar spinal stenosis by PES, adequate exposure and surgical field expansion should be achieved by adjusting the position of the working channel. The herniated intervertebral disc tissues should be completely resected. Moreover, it should be also checked whether there are loose but non-herniated nucleus pulposus tissues in the intervertebral disc and intervertebral space. If yes, they should be resected completely as well to reduce the probability of relapse. However, for degenerative intervertebral disc bulges with intact structure and mild compression, the surgical focus is placed on removing the compression-inducing factors in the posterior and lateral sides of the spinal canal, without impairing the integrity of the intervertebral disc.^10^
5) Inadequate decompression: inadequate decompression occurred in 7 out of 19 patients undergoing a second surgery with all three approaches. It was the most common reason for a second surgery following PES for lumbar spinal stenosis. Reasons for inadequate decompression: varying sources of patients for a second surgery; unskilled manipulations by some surgeons, with inaccurate intraoperative judgment and inadequate exploration causing incomplete removal of compression-inducing factors, especially in early-stage patients; poor position of the working channel or difficulty of adjusting the working channel, leading to difficult decompression in key sites, including lateral recess; multiplicity of bleeding points and difficulty of providing hemostasis; this is especially true for PIED, where the number of bleeding points is larger and thorough hemostasis may be difficult. As a result, the endoscopic surgical field is blurred, and adequate decompression is almost impossible; immature termination of surgery for some reasons, as in patients who cannot endure hours-long surgery or dural sac tear. From the above facts, the following conclusions can be drawn: The pathogenic factors of lumbar spinal stenosis are more complicated than those of LIDH. For the former, the culpable site of stenosis is more extensive, and there is a wider range of pathogenic factors, including hyperplastic sclerotin, thickened ligamentum flavum, and herniated intervertebral disc. During surgery, all these sites need to be adequately decompressed, otherwise the efficacy will be impaired. An appropriate approach to PES should be chosen based on the specific features of stenosis. For example, decompression may be difficult with PTED for severe stenosis in the lateral and dorsal aspects or bony stenosis, and this procedure should be chosen with caution.

### Precautions and choice of indications for PES for lumbar spinal stenosis

Precautions: The treatment of lumbar spinal stenosis by PES is different from lumbar disc herniation. Due to a greater lesion, the manipulations are more difficult, and the skill requirements are higher. 1) The culpable segments, primary pathogenic factors and decompression should be determined according to preoperative symptoms, signs and radiological data. 2) Surgical manipulations should be standardized to avoid nerve injury. Local anesthetics or neurological monitoring can be performed to timely assess the patients’ conditions and provide appropriate treatment. 3) The surgical approach should be chosen according to the culpable segments; 4) Secondary foraminoplasty can be performed under the endoscope if foraminoplasty is inadequate with PTED; or, the hyperplastic sclerotin in the ventral side of the articular process can be drilled away under the endoscope to enlarge the operational space. 5) Timely and effective hemostasis should be provided to ensure a clear endoscopic surgical field and to avoid damage to the nerve and neural sac, through the following steps: adjust water pressure, use hemostatic drugs, radiofrequency ablation and coil. 6) Remove the loosened nucleus pulposus tissues to avoid another dislocation. 7) Before ending the surgery, the position of the working channel should be adjusted with careful observation in all directions, so as to avoid residual compression or nerve-entrapping bone fragments. 8) Radiofrequency ablation can be performed during the withdrawal from the working channel to provide sufficient hemostasis and to avoid hematoma. 9) Surgeons should choose the most familiar approach, and an intraoperative shift or staged operation is recommended if necessary.

Choice of surgical indications and approach: PES has the benefits of less invasiveness and fast recovery for the treatment of lumbar spinal stenosis. Nevertheless, good outcomes can be achieved only with the appropriate choice of surgical indications and adequate decompression. In the present study, 23 patients underwent a second surgery following PES, which indicated the limitation of PES for treating lumbar spinal stenosis. So far, PES cannot be used as a substitute for MED or MMED. There are generalized rules for the choice of surgical approach, and decisions shall be made based on specific conditions. First, PTED achieved spinal canal decompression via foraminoplasty and it is a convenient procedure for treating unilateral lateral spinal canal stenosis or intervertebral foramen stenosis. PTED is more favored when lumbar spinal stenosis is combined with significant LIDH. While decompressing the lateral spinal canal, the herniated intervertebral disc can be resected to achieve better outcomes. Second, posterior PIED or PIED using the Delta endoscope is more favored for lumbar spinal stenosis featured by lateral hyperplasia but insignificant LIDH, as this procedure can achieve direct decompression.^11^^,^^12^ However, for lumbar spinal stenosis combined with lumbar instability and unstable spondylolisthesis, PES may further impair lumbar stability and aggravate the symptoms and hence should be used with caution. In a word, an appropriate surgical approach should be chosen depending on the specific features of the culpable segments in lumbar spinal stenosis, so as to achieve the best outcomes while causing less damage and invasiveness.

### Limitations of the present study

In the present study, there were 19 patients receiving a second surgery, among which 13 patients had previously received PES at other hospitals. Therefore, surgical indications and skills varied greatly among surgeons performing the primary surgery on these patients. This definitely affected surgical outcomes and the incidence of the second surgery. Secondly, there were large variations in postoperative follow-up. Some patients were only followed up for a short period. The symptoms could be temporarily relieved with partial decompression, removal of some inflammatory mediators from the lesion site along with the water medium in PES, and reduction of neural stimuli. However, there is still a risk of relapse or symptom aggravation over time for these patients and that means the number of patients requiring a second surgery will increase. Thirdly, those who did not undergo a second surgery at the studied department despite the poor outcomes after the primary treatment or later received a second surgery at other hospitals were excluded. Finally, the sample size included in this study was small, and this was a retrospective study rather than a prospective controlled study. It did not rule out some anatomical and physiological variables on the effect of endoscopic spinal decompression interference.

Severe hyperplasia, obscure anatomic structure, lumbar instability, and nerve injury are the common reasons for a second surgery after PES for lumbar spinal stenosis. Appropriate indications and surgical approaches can be chosen based on the patient's situations and technical conditions. Subject to the quantitative and qualitative limitations of the included studies, the above conclusions need to be validated by more high-quality studies.

## Availability of data and materials

The datasets used and/or analyzed during the present study are available from the corresponding author upon reasonable request.

## Ethics approval

The present study was approved by the Ethics Committee of Ningbo No.6 Hospital (n° 201409NB23) and written informed consent was provided by all patients prior to the study start. All procedures were performed in accordance with the ethical standards of the Institutional Review Board and The Declaration of Helsinki, and its later amendments or comparable ethical standards.

## Authors’ contributions

LianSong Lu designed the research study. LianSong Lu and ZhenShan Yuan performed the research. HaoJie Li and ShaoHua Sun provided help and advice on the experiments. ZhenShan Yuan and HaoJie Li analyzed the data. LianSong Lu wrote the manuscript. All authors contributed to editorial changes in the manuscript. All authors read and approved the final manuscript.

## Declaration of competing interest

The authors declare no conflicts of interest.
